# Highly stretchable and reliable graphene oxide-reinforced liquid gating membranes for tunable gas/liquid transport

**DOI:** 10.1038/s41378-020-0159-x

**Published:** 2020-07-13

**Authors:** Wei Lv, Zhizhi Sheng, Yinglin Zhu, Jing Liu, Yi Lei, Rongrong Zhang, Xinyu Chen, Xu Hou

**Affiliations:** 1 Department of Physics, Research Institute for Biomimetics and Soft Matter, Fujian Provincial Key Laboratory for Soft Materials Research, Jiujiang Research Institute, College of Physical Science and Technology, Xiamen University, 361005 Xiamen, China; 2 State Key Laboratory of Physical Chemistry of Solid Surfaces, College of Chemistry and Chemical Engineering, Xiamen University, 361005 Xiamen, China; 3 Collaborative Innovation Center of Chemistry for Energy Materials, Xiamen University, 361005 Xiamen, China

**Keywords:** Materials science, Engineering

## Abstract

The ability of membrane technologies to dynamically tune the transport behavior for gases and liquids is critical for their applications. Although various methods have been developed to improve membrane success, tradeoffs still exist among their properties, such as permeability, selectivity, fouling resistance, and stability, which can greatly affect the performance of membranes. Existing elastomeric membrane designs can provide antifracture properties and flexibility; however, these designs still face certain challenges, such as low tensile strength and reliability. Additionally, researchers have not yet thoroughly developed membranes that can avoid fouling issues while realizing precise dynamic control over the transport substances. In this study, we show a versatile strategy for preparing graphene oxide-reinforced elastomeric liquid gating membranes that can finely modulate and dynamically tune the sorting of a wide range of gases and liquids under constant applied pressures. Moreover, the produced membranes exhibit antifouling properties and are adaptable to different length scales, pressures, and environments. The filling of graphene oxide in the thermoplastic polyurethane matrix enhances the composites through hydrogen bonds. Experiments and theoretical calculations are carried out to demonstrate the stability of our system. Our membrane exhibits good stretchability, recovery, and durability due to the elastic nature of the solid matrix and dynamic nature of the gating liquid. Dynamic control over the transport of gases and liquids is achieved through our optimized interfacial design and controllable pore deformation, which is induced by mechanical stimuli. Our strategy will create new opportunities for many applications, such as gas-involved chemical reactions, multiphase separation, microfluidics, multiphase microreactors, and particulate material synthesis.

## Microsystems & Nanoengineering Fluidics: Tuning into better performing membranes

Chinese scientists have developed a tunable membrane that is highly stretchable and could be used in a range of applications, including microfluidic devices, multiphase microreactors, and for particulate material synthesis. The ability to dynamically tune the transport behavior of gases and liquids is critical for membrane applications. And although recent tunable elastomeric membrane designs offer flexibility and anti-fracture properties, they have low tensile strength, are unreliable, and suffer from fouling. Now, Xu Hou and colleagues from Xiamen University in China use a novel liquid gating technology to develop a membrane made from graphene oxide-reinforced thermoplastic polyurethane that can be dynamically tuned for a wide range of gases and liquids, while also exhibiting anti-fouling properties. Liquid gating technology which uses a capillary-stabilized functional liquid to form reversible gates inside the pores, shows prominent properties in controlling complex, selective, multiphase substance transport. The liquid gating membrane is adaptable to different length scales, pressures, and environments and could be used in multiphase separation, chemical reactions, and drug delivery.

## Introduction

The ability to dynamically tune the gating and transport behavior of gases and liquids is useful in the operation of membrane-based systems for various applications, such as multiphase separation^1–4^, drug delivery^5^, and chemical reactions^6^. Recently, the mechanism of liquid gating membranes has opened up possibilities to enable selective transport and complex sorting of multiphase fluids within a single system. This unified liquid gating strategy utilizes a capillary-stabilized liquid to form a reconfigurable gate inside the pores of membranes, and because the critical pressures required to open the gate are differential for gases and liquids, the separation of target substances can be obtained. During this process, the liquid lining in the membrane would lead to molecular smooth surfaces with the advantages of lower transmembrane pressures for liquids and sustainable antifouling behavior^7,8^. In addition, the pore size of membranes is a key factor in determining membrane performance, such as permeability and selectivity^7,9,10^. With the liquid gating strategy, the change in the pore sizes can also cause a responsive change in the critical pressures for transport substances^11,12^. Therefore, with adjustable pore sizes, dynamic control over the gating and transport behavior for multiphase transport can be realized even under one constant pressure^13^. The application of tension (i.e., stretching) can easily lead to the deformation of an elastomeric membrane, thereby resizing the pores. Therefore, a liquid gating elastomeric porous membrane system could be useful in applications requiring dynamic multiphase transport and separation under steady-state pressures^12^. However, improving the success of liquid gating elastomeric membrane systems requires membranes with better materials and performance. For example, elastomeric membranes with high tensile properties and high reliability still require vigorous development.

As a widely applied elastomeric material, thermoplastic polyurethane (TPU) is a versatile multiblock synthetic polymer with customizable properties, which can be tuned by adjusting the constituent monomeric materials depending on requirements^14^. However, the stiffness and mechanical strength of TPU are relatively low due to the lower composition of hard segments, resulting in its limited application^15^. Incorporation of fillers, such as carbon nanotubes^16,17^ and graphene^18^, into the TPU matrix can enhance the physicochemical properties of TPU composites. Among these various composites, graphene oxide (GO)-doped TPU composites have drawn significant attention from researchers^19–23^ due to their unique combination of properties^24^.

In this study, we introduce a highly stretchable and reliable graphene oxide-reinforced TPU liquid gating elastomeric porous membrane (GO/TPU LGEPM) system, which has high durability and superior antifouling properties. We demonstrate the design strategy of the GO/TPU LGEPM from material preparation and interfacial properties to the working principle for tunable gas/liquid transport. With GO as a filler in the TPU matrix, GO/TPU composites exhibit enhanced mechanical properties that are attributed to the hydrogen bond between GO and TPU. In addition, by infiltrating with the functional gating liquid, a stable GO/TPU liquid gating system is established on the basis of the interfacial design and wetting properties. The liquid gating mechanism also endows GO/TPU LGEPM with sustainable antifouling behavior for transporting liquid with the liquid–liquid interface. The transmembrane transport behavior of gases and liquids through the GO/TPU LGEPM and the stress-responsive characteristics were also demonstrated. The tunable gas/liquid transport of the GO/TPU LGEPM is achieved by stretching and releasing, which shows superior durability after long-term operation. This system promotes applications in gas-involved chemical reactions and multiphase separation and can be further exploited for other more complicated smart membrane materials.

## Results and discussion

### Design of the graphene oxide-reinforced liquid gating membranes

**Fig. 1:**
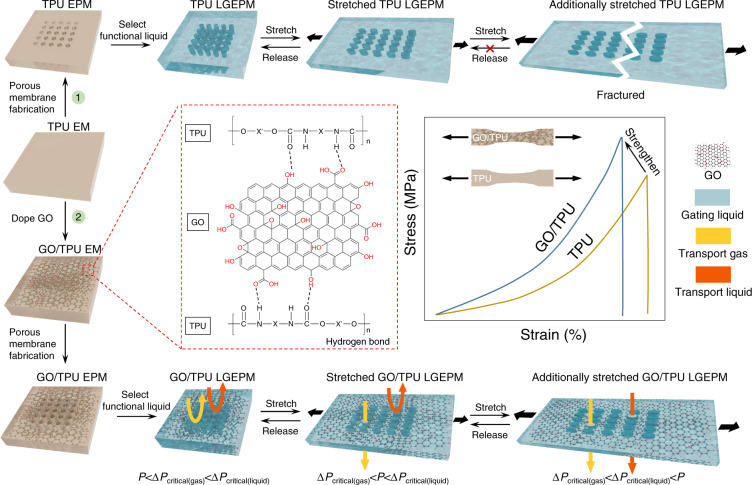
Schematic illustration of the fabrication process of the GO/TPU LGEPM. The tensile strength of the GO/TPU EM is enhanced by doping GO into the TPU matrix due to the hydrogen bonds between the oxygen groups of GO and the N-H groups of TPU. X and X′ represent the aromatic compounds. Number 1 represents the fabrication of the TPU porous membrane, whereas number 2 represents the fabrication of the GO/TPU membrane. The GO/TPU elastomeric porous membrane (GO/TPU EPM) is fabricated from the GO/TPU EM by laser cutting. The GO/TPU LGEPM is formed by infiltrating the gating liquid into the GO/TPU EPM. The transport of gases and liquids can be dynamically controlled by the deformation of the GO/TPU LGEPM. When both Δ*P*_critical(gas)_ and Δ*P*_critical(liquid)_ are above the applied pressure *P*, neither gas nor liquid will pass through the GO/TPU LGEPM. The pore size of the membrane increases, leading to a drop in the critical pressures for transporting substances. When Δ*P*_critical(gas)_ is below *P* and Δ*P*_critical(liquid)_ is above *P*, only gas permeates the system. Upon further stretching, the critical pressures continue to drop. If both Δ*P*_critical(gas)_ and Δ*P*_critical(liquid)_ are below *P*, both gas and liquid will permeate the system. Once the stress is released, the GO/TPU LGEPM can recover to its initial state

Figure 1 illustrates the design strategy and operation mechanism of the GO/TPU LGEPM. The GO/TPU elastomeric membrane (GO/TPU EM) was prepared by a solution blending method (Fig. S1), which can form hydrogen bonds between the oxygen groups from GO and the N–H groups from TPU^25,26^. The hydrogen bonds significantly enhance the tensile strength of the composites (Fig. 1). With a laser cutting technique, the porous structures on the membranes can be carefully made for fabricating the GO/TPU elastomeric porous membrane (GO/TPU EPM) (Fig. S1). Then, to establish a liquid gating system with high-performance antifouling properties, the GO/TPU EPM was infiltrated with a functional gating liquid to create the GO/TPU LGEPM. Compared with pure TPU LGEPM, the GO/TPU LGEPM exhibits a superior tensile strength, which allows it to reversibly withstand cycles of stretching and releasing. Moreover, the infused gating liquid can have a random shape that adjusts to the applied pressure, thereby providing an ideal dynamic material to solid porous membranes for regulating gas/liquid transport. According to the stable interfacial design^27^, the membrane matrix and the infiltrated gating liquid should be matched for a lower energy state to ensure the stability of this liquid gating system. Silicone oil was chosen as the gating liquid in our demonstration, which is discussed hereafter.

Under mechanical stress, the prepared GO/TPU LGEPM can be stretched, leading to the expansion of the pores. Due to the size effect, the critical pressures required for transport substances can change^11,27^. Therefore, the critical pressures of the transport substances can be dynamically controlled through deformation by stretching and releasing the GO/TPU LGEPM (Fig. 1). In contrast to conventional membranes in which gases can passively pass through, in a liquid gating membrane system, both gases and liquids need to overcome critical pressures to cross the membrane. Based on the above mechanism, the mixture of gas and liquid can be separated effectively by the GO/TPU LGEPM. For example, when the applied pressure (*P*) is lower than the critical pressure of the gas (Δ*P*_critical(gas)_) and liquid (Δ*P*_critical(liquid)_), neither phase could pass through the GO/TPU LGEPM (represented as *P* < Δ*P*_critical(gas)_ < Δ*P*_critical(liquid)_). Then, when the GO/TPU LGEPM is stretched uniaxially, the pore size increases in the direction of the stretch. As a result, the critical pressures for the gas and liquid decrease until *P* is higher than Δ*P*_critical(gas)_ but lower than Δ*P*_critical(liquid)_ (represented as Δ*P*_critical(gas)_ < *P* < Δ*P*_critical(liquid)_). In this way, the gas is permitted to pass through the GO/TPU LGEPM, while the transport liquid remains blocked. When a uniaxial stretch is further applied to the GO/TPU LGEPM, the pore size is continuously increased, resulting in a further decrease in the critical pressures of the transport substances. The mixture of transport liquid and gas permeates the GO/TPU LGEPM when *P* surpasses both Δ*P*_critical(gas)_ and Δ*P*_critical(liquid)_ (represented as Δ*P*_critical(gas)_ < Δ*P*_critical(liquid)_ < *P*). Then, after the tension is released, the GO/TPU LGEPM returns to its initial state.

### Structural characterization and mechanical properties of the GO/TPU EM

**Fig. 2:**
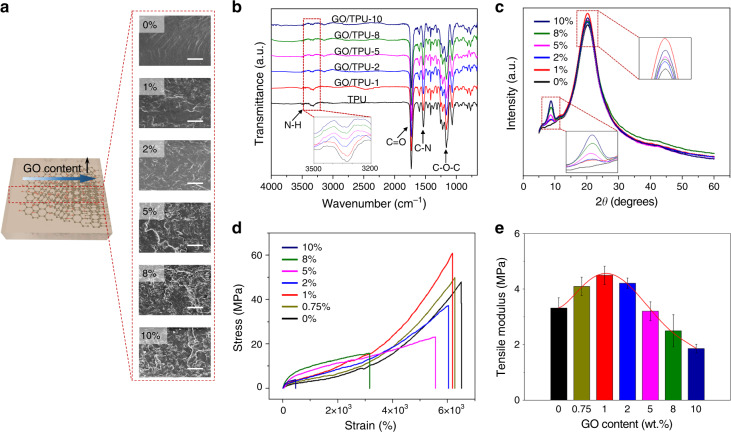
Structural characterization and mechanical properties of the GO/TPU EM. **a** Schematic and surface morphology of the GO/TPU EM with different GO contents, wherein the scale bar is 25 μm. **b** FTIR spectra of the GO/TPU EM. TPU composites with 0 to 10 wt.% GO were designated TPU, GO/TPU-1, GO/TPU-2, GO/TPU-5, GO/TPU-8, and GO/TPU-10 according to the filler concentration. **c** X-ray diffraction pattern of the GO/TPU EM at a scanning rate of 4°/min. **d** Stress-strain curves of the GO/TPU EM. The membranes were stretched at a constant displacement rate of 50 mm/min at room temperature. **e** Effect of filler content on the tensile strength of the GO/TPU EM

The morphology of GO was observed by scanning electron microscope (SEM), and the thickness was determined by AFM (Fig. S2a–d). In Fig. S2c, d, the thickness is 2.5 nm. The optical images of GO/TPU composites with different GO contents are shown in Fig. S3. The morphology of the GO/TPU composites was observed by SEM. For the SEM images shown in Fig. 2a, different amounts of GO (1 wt.% to 10 wt.%) are dispersed in the TPU matrix of the samples. With the increase in GO content, GO may aggregate in the TPU matrix, thereby impairing the mechanical properties of the GO/TPU EM. A good dispersion is formed due to the strong interfacial interaction (hydrogen bond) between the GO and TPU and the enhanced bonding between the GO and TPU from fast solvent evaporation by using a hot press technique.

The formation of the GO/TPU EM and the chemical interaction between the GO and TPU were determined by Fourier transform infrared (FTIR) spectroscopy. The FTIR spectroscopy results of the TPU and GO/TPU composites are shown in Fig. 2b. As a comparison, the spectroscopy results of GO are displayed in Fig. S4. The presence of characteristic bands at 3327 cm^−1^ (hydrogen-bonded N–H stretching), 1733 cm^−1^ (non-hydrogen bonded or free C=O stretching), 1532 cm^−1^ (C–N stretching), and 1221 cm^−1^ (C–O–C) confirmed the formation of urethane linkage, –NH–C(O)–O–, in TPU and its composites. As shown in the inset of Fig. 2b, the peak representing the stretching vibration of hydrogen-bonded N–H groups shifts from 3327 cm^−1^ for pristine TPU to 3330 cm^−1^ for the composite with 5 wt.% GO, and then shifts to 3333 cm^−1^ as the GO content increases to 10 wt.%. This 3–6 cm^−1^ redshift of the hydrogen-bonded N–H band indicates a reduction in the average strength of the internal hydrogen bonding in TPU. This reduction is attributed to the formation of a new hydrogen bond between N–H in TPU and the oxygen functional group in GO, resulting in the interruption of the N–H/C = O hydrogen bond in TPU^28–32^.

XRD spectra of the pure TPU and GO/TPU composites are shown in Fig. 2c. TPU is a soft elastomeric material^33–35^ with a broad peak at 2*θ* of 20.3°, which is associated with the (110) crystal plane of soft segments in TPU. As shown in the inset of Fig. 2c, the peak intensity at a 2*θ* of 20.3° increases with increasing GO loading, whereas the peak position remains the same. This clearly verifies that the crystallinity of the GO/TPU composites in the amorphous region increased with increasing filler concentration^20^. We observed that the GO filler had the greatest effect on the crystallinity of the TPU composite when the GO content was 1 wt.%. This finding may be attributed to the combined influence of a stronger nucleation effect and less microphase separation between hard and soft domains induced by GO^36^. For another peak at 2*θ* of 8.9° shown in the inset of Fig. 2c, this new (002) diffraction peak is assigned to the GO-induced crystallization^35,37^. The peak intensity increases with increasing GO content, indicating that the crystalline structure, which is crucial in maintaining the mechanical properties of GO/TPU composites, is not obviously destroyed by the addition of GO. The d-spacing and crystal size of each peak that appeared in the GO/TPU composites are shown in Table S1.

Figure 2d shows the stress-strain curves of the pure TPU and GO/TPU composites. Pure TPU exhibits a high tensile strength (46.8 MPa). The tensile strength decreases with the increase in the GO content except for GO/TPU-1, for which the tensile strength increases to 50.5 MPa. The main reason for the increase in tensile strength is the small increase in GO content, which can promote strain hardening. At a higher GO content, the decrease in tensile strength is due to the inhibition of molecular rearrangement and orientation with respect to the tensile axis^21^, which might be related to the phase separation between GO and TPU that deteriorates the mechanical properties. Moreover, the tensile modulus of the composites increases as the GO content increases (Fig. 2e), reaching the greatest value when the GO content is 1 wt.%. Hydrogen bonding, covalent bonding and polar-polar interactions between graphene oxide and TPU chains can stiffen the hard domain of TPU, thereby increasing the tensile modulus of the composites at a lower content^38^. The stress-strain test results are consistent with the results obtained from the XRD study, which shows a good dispersion of 1 wt.% GO within the TPU matrix. In the following measurements, we choose GO/TPU composites with 1 wt.% GO as the substrate of the GO/TPU LGEPM.

### Stability and antifouling properties of the GO/TPU liquid gating membranes

**Fig. 3:**
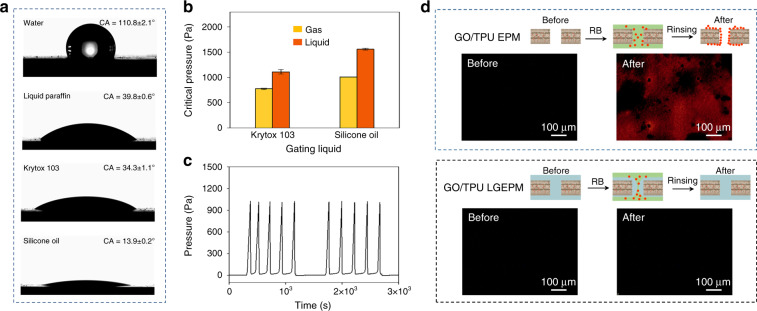
Stability and antifouling properties of the GO/TPU LGEPM. **a** The wettability of the GO/TPU EM. The CA of water, liquid paraffin, Krytox 103, and silicone oil was tested on a solid elastomeric membrane. **b** Critical pressures of gas and liquid flowing through the GO/TPU LGEPM in different gating liquids. **c** Cyclic tests of gas flowing through the GO/TPU LGEPM with silicone oil as the gating liquid. **d** Antifouling properties of the GO/TPU LGEPM in comparison to the GO/TPU EPM. The fluorescence images show the GO/TPU EPM and silicone oil-infused GO/TPU LGEPM before and after rhodamine B treatment

The strong affinity between the gating liquid and membrane materials to avoid replacement by transport liquid is critical to the stability of a liquid gating system. Therefore, the wettability of the gating and transport liquid on the GO/TPU EM, the interfacial energy, and the stability of different configurations have been investigated. The contact angle (CA) of water, liquid paraffin, Krytox 103 lubricant oil (K103), and silicone oil on the substrates of GO/TPU EM were measured (Fig. 3a). Water has a CA of 110.8 ± 2.1° on the GO/TPU EM. Liquid paraffin and K103 have CA values of 39.8 ± 0.6° and 34.3 ± 1.1° on the GO/TPU EM, respectively. Silicone oil has a lower CA of 13.9 ± 0.2° on the GO/TPU EM. Distinctly, K103 and silicone oil preferentially wet the substrate better than water. However, the apparent CA is insufficient to demonstrate the overall stability of the system. Thus, it is necessary to measure the surface tension of three liquids and the interfacial surface tension between water and these liquids (Table 1). To verify the stability of the GO/TPU LGEPM system, the total interfacial energies of the following configurations are calculated^12^: (1) the membrane is infiltrated with the gating liquid on which the transport liquid floats (*E*_1_), (2) the membrane is infiltrated with the gating liquid (*E*_2_), and (3) the membrane is infiltrated with the transport liquid (*E*_3_). To ensure that the GO/TPU EPM has a higher affinity to the gating liquid than the transport liquid^39^, the following conditions should be satisfied: ${\mathrm{\Delta }}E_{\text{I}} = E_{3} - E_{1}\,>\, 0$ and ${\mathrm{\Delta }}E_{\mathrm{II}} = E_{3} - E_{2}\,>\, 0$. Note that Δ*E*_I_ and Δ*E*_II_ are interpreted as

1 $${\mathrm{\Delta }}E_{\mathrm{I}} = R\left( {\gamma _B{\mathrm{cos}}\theta _B - \gamma _A{\mathrm{cos}}\theta _A} \right) - \gamma _{AB}$$

2 $${\mathrm{\Delta }}E_{{\mathrm{II}}} = R\left( {\gamma _B{\mathrm{cos}}\theta _B - \gamma _A{\mathrm{cos}}\theta _A} \right) + \gamma _A - \gamma _B$$

where *R* is the roughness factor of the GO/TPU EPM (*R* = 2), which is determined by the ratio between the actual and projected surface areas; *γ*_A_, *γ*_B_, and *γ*_AB_ represent the surface tension of the transport liquid, gating liquid, and the interfacial surface tension between the transport and gating liquids, respectively; and *θ*_A_ and *θ*_B_ are the equilibrium contact angles of the transport and gating liquids on a flat GO/TPU EM surface, respectively. Theoretically, when both Δ*E*_I_ and Δ*E*_II_ are positive values, the GO/TPU LGEPM is a stable system. In contrast, if both are negative values, the GO/TPU LGEPM tends to be unstable. Based on the energy calculations in Table 1, infusing liquid paraffin, silicone oil, and K103 into the GO/TPU elastomeric porous membranes ensures that the GO/TPU LGEPM is theoretically stable for transporting deionized (DI) water.

**Table 1:** Comparisons between theoretical and experimental results for different gating liquid/transport liquid combinations

Case no.	Transport liquid (A)	Gating liquid (B)	*γ*_A_	*γ*_B_	*γ*_AB_	*θ*_A_	*θ*_B_	Δ*E*_I_	Δ*E*_II_	Stable system	
										Theo.	Exp.
1	DI water	Liquid paraffin	72.7	29.5	41.5	110.8	39.8	55.5	140.2	Y	Y
2	DI water	Krytox 103	72.7	17.0	53.6	110.8	34.3	26.1	135.4	Y	Y
3	DI water	Silicone oil	72.7	17.5	42.5	110.8	13.9	43.1	140.8	Y	Y
4	Liquid paraffin	Silicone oil	29.5	17.5	0.5	39.8	13.9	−11.9	0.6	Y/N	N
5	Liquid paraffin	Krytox 103	29.5	17.0	1.2	39.8	34.3	−18.4	−4.7	N	N
6	Silicone oil	Krytox 103	17.5	17.0	9.6	13.9	34.6	−15.6	−5.5	N	N

The parameters were measured at room temperature (25 °C). The units of γ_A_, γ_B_, γ_AB_, Δ*E*_I_, and Δ*E*_II_ are mN/m, whereas those of *θ*_A_ and *θ*_B_ are degrees (°)

To further investigate the stability of different liquid gating systems, K103 and silicone oil were used as the gating liquids, which show a stronger affinity between the gating liquid and the substrate, while water was used as the transport liquid. In Fig. 3b, with silicone oil as the gating liquid, the critical pressures of the gases and liquids are higher than those when using K103 as the gating liquid. Moreover, the pressure difference between the gases and liquids in the silicone oil-infused GO/TPU EPM is much larger than that in the K103-infused GO/TPU EPM. As shown in Fig. 3c, the critical pressure for flowing gas is stable over a long period of time when using silicone oil as the gating liquid. As previously mentioned, silicone oil was chosen as the gating liquid, and water was taken as the transport liquid for further demonstration due to the stability of this configuration both theoretically and experimentally. Moreover, fouling experiments were conducted on both the GO/TPU EPM and GO/TPU LGEPM by flowing rhodamine B (RB) solution through the membranes (Fig. 3d). The reason for choosing RB as a representative example to test the antifouling properties is due to the extremely sticky nature of RB, which can attach to surfaces by nonspecific binding. Both the GO/TPU EPM and the GO/TPU LGEPM were treated with RB solution and then extensively rinsed with DI water. Before the treatment, neither the GO/TPU EPM nor the GO/TPU LGEPM showed any fluorescence. After RB treatment, the GO/TPU EPM was contaminated by RB even after extensive rinsing, whereas the GO/TPU LGEPM does not show any fluorescence, indicating its good antifouling properties. The good antifouling performance is attributed to the existence of a stable liquid–liquid interface between the gating liquid and the RB solution, which is contrasted by the easily contaminated solid surface present in other cases.

### Stress-responsive membrane transport behavior

**Fig. 4:**
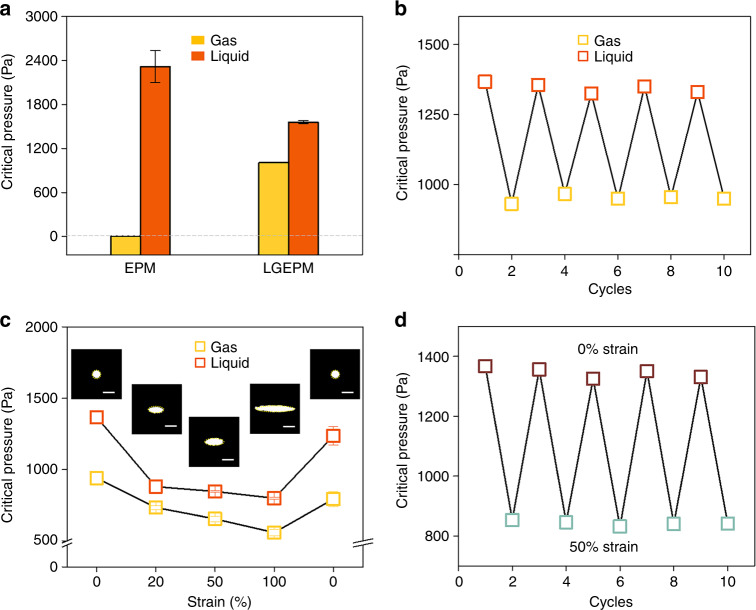
Stress-responsive transmembrane transport behavior of the GO/TPU LGEPM. **a** Transmembrane critical pressures of gases and liquids flowing through the GO/TPU EPM and GO/TPU LGEPM. Note that the error bars indicate SD and *N* = 3. **b** Cyclic tests of the gas and liquid passing through the GO/TPU LGEPM. **c** Transmembrane critical pressures of gas and liquid flowing through the GO/TPU LGEPM as a function of one-dimensional strain. The insets are optical images of the GO/TPU LGEPM during the stretching and releasing process. Note that the scale bar is 100 μm, the error bars indicate SD, and *N* = 3. **d** Transmembrane critical pressure of liquid flowing through the GO/TPU LGEPM in response to alternate zero strain and 50% strain

To characterize the transmembrane behavior of fluids in stress-responsive membrane systems, the critical pressures of the fluids required to pass through the GO/TPU EPM and GO/TPU LGEPM were measured under the same operating conditions. First, the flow rate of the transport substances can affect the critical pressures and the stability of the membrane system^11,12^. Comparing the critical pressures of gases and liquids flowing through the membrane at different flow rates, a flow rate of 500 μL/min was used for more experiments (Fig. S5). As shown in Fig. 4a, there are substantial differences between the critical pressures of the gas and liquid when flowing through the GO/TPU EPM with and without the gating liquid. The transmembrane critical pressure for liquid transport is in the range of 2318 ± 218 Pa for the GO/TPU EPM. Compared to the bare membrane, the GO/TPU LGEPM can reduce the transmembrane pressure of liquid transport by 32% with a value of 1560 ± 18 Pa, indicating energy-saving properties. The cyclic stability of the gas and liquid transport passing through the GO/TPU LGEPM is shown in Fig. 4b. The key feature that Fig. 4a, b demonstrates is an advantage of the liquid gating mechanism of the GO/TPU LGEPM to create tunable critical pressures for both gas and liquid transport and enables energy-saving properties for liquid transport.

The pore size is a key factor in determining the critical pressures of transport substances^11,12,27^. Furthermore, according to the elastic properties of the GO/TPU LGEPM, the pore geometry can be adjusted dynamically. The gating liquid held by the solid porous matrix can also form a random shape that is adjusted to the applied pressure. Thus, the critical pressures of the transport substances through this system could be dynamically tuned by the coordinated effect of the pore size deformation and the fluidity of the liquid lined in the pore. The size of the pore increases during uniaxial stretching in the inset of Fig. 4c, whereas the critical pressures of gases and liquids both decrease with increasing strain. To verify the change in the pore size during the deformation of the membrane, the simulation of pore deformation was calculated. The equivalent stress distribution of the 4 × 4 array GO/TPU EPM is shown in Fig. S6. When the system is stretched to 100% strain, Δ*P*_critical(gas)_ decreases from 939 ± 48 to 557 ± 22 Pa, while Δ*P*_critical(liquid)_ reduces from 1366 ± 44 to 799 ± 7 Pa (Fig. 4c). When the stress is released, the GO/TPU LGEPM returns to its original state. To further examine this behavior, the critical pressure of the liquid in 10 cycles of stretching and releasing (50% strain) was measured to verify the stability of this system (Fig. 4d). The critical pressure of the liquid changes periodically during stretching and releasing, following the principle of reducing when the GO/TPU LGEPM is stretched and recovering when the mechanical force is released (Fig. 4c, d). As previously mentioned, the GO/TPU LGEPM has a real-time pressure response to the mechanical stimulus, and each transport substance has a specific critical pressure to pass through the membrane. Hence, this dynamic system paves the foundation for the dynamic transportation and separation of different fluids.

### Tunable gas/liquid transport of the GO/TPU LGEPM

**Fig. 5:**
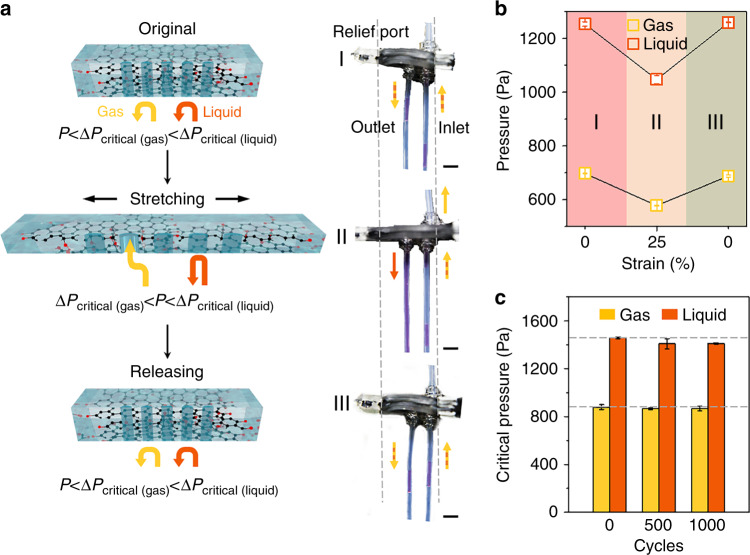
Tunable gas/liquid transport of the GO/TPU LGEPM by stretching and releasing. **a** Scheme description and snapshots of the GO/TPU LGEPM under various strains. **b** Pressure control for separating gas and liquid in a dynamic strain process. Note that the scale bar is 2 cm. **c** The durability of the GO/TPU LGEPM. The pressures for gas and liquid transport were measured after the cyclic stretching (50% strain) and releasing process

According to the results shown above, the critical pressures for transporting gas and liquid are dynamically controlled by stretching and releasing the GO/TPU LGEPM, while the applied pressure *P* remains constant during the deformation. Gas and liquid can be effectively separated from the mixture by the GO/TPU LGEPM (Figs. S7 and  5a). In the original state (represented as *P* < Δ*P*_critical(gas)_ < Δ*P*_critical(liquid)_), both gas and liquid were blocked due to the small pore size and high critical pressures (Fig. 5a, state I). When the critical pressure of gas falls below the applied pressure due to the increase in pore size induced by the stretching process (represented as Δ*P*_critical(gas)_ < *P* < Δ*P*_critical(liquid)_), gas penetrates the membranes to the relief port while liquid flows through the outlet (Fig. 5a, state II). Thus, the outlet is degassed liquid. In this case, gas and liquid could be thoroughly separated. On the basis of our design, the separation efficiency is ~98.1%. When the stress is released, both gas and liquid are blocked by the GO/TPU LGEPM and flow through the outlet (Fig. 5a, state III). The whole process of tunable gas and liquid transport is determined by measuring the critical pressures of the gas and liquid, as shown in Fig. 5b. It is worth mentioning that this approach realizes phase transport and separation by simply dynamically adjusting the mechanical stress.

To further explore the high stretchability and excellent reliability of the GO/TPU LGEPM, the critical pressures of the transport gases and liquids during multiple cycles of stretching and releasing of the GO/TPU LGEPM were measured (Fig. 5c). The critical pressures were measured every 500 cycles of 1000 cyclic stretches and releases. Compared with the original state, the critical pressure of the gas, which has undergone 1000 cycles of stretching and releasing, is reduced from 878 ± 7 Pa to 866 ± 5 Pa, and the critical pressure of the liquid is reduced from 1457 ± 21 Pa to 1410 ± 19 Pa. The stable pressure values indicate that the GO/TPU LGEPM has splendid stability in the cyclic stretching and releasing process.

## Conclusions

In this study, we prepared graphene oxide-reinforced liquid gating membranes with enhanced mechanical properties as a stress-responsive liquid gating system that can dynamically control the transport and separation of gas and liquid in response to a mechanical stimulus under a steady-state applied pressure. The structural characterization and mechanical properties of the GO/TPU composites were determined, and the GO filler enhanced the tensile strength of the GO/TPU composites by hydrogen bonding between the GO and the TPU polymer. The performance metrics of the systems, such as selectivity, stability, and antifouling properties, were also investigated. To ensure the stability of the system, we experimentally and theoretically investigated different material configurations. With an optimized material selection and interfacial design, the system exhibited superior antifouling behavior. In addition, we also established distinct transmembrane threshold profiles of the GO/TPU LGEPM for transporting gases and liquids. By simply alternating stretching and releasing processes, a responsive real-time pressure variation for transporting gases and liquids was achieved. Moreover, a wider range of substance transport and separation was achieved by mechanically adjusting the extent of stretching on the membrane system. The superior durability of the GO/TPU LGEPM ensures the long-term stability of the threshold pressures of gases and liquids. We anticipate that the high stretchability, dynamic tunability, and reliability of the GO/TPU LGEPM combined with the liquid–liquid or liquid–gas interfacial design will benefit fields ranging from smart gating membranes, gas-involved chemical reactions, multiphase separation, microfluidics, to particulate material synthesis and beyond.

## Materials and methods

### Materials and reagents

GO was provided by the Institute of Coal Chemistry, Chinese Academy of Sciences. The GO was prepared by a modified Hummers’ method^40,41^. As a precursor, the as-prepared GO was ground into fine powders (~100 mesh) and further air-dried at 100 °C for 3 h. This material was then loaded into a quartz tube, for which one end was sealed while the other end was connected to a vacuum pump. The tube was evacuated to a pressure of <2.0 Pa, and then a heating schedule was executed with a heating rate of 30 °C/min. At ~200 °C, an abrupt expansion of GO was observed, generating mass fluffy black powder. The as-fabricated GO has 72.18 at.% C and 27.82 at.% O with a C/O atomic ratio of 2.59. GO contains functional groups, such as –OH and –COOH^41^. Thermoplastic polyurethane (TPU, Elastollan Soft 60A, polyester, 1.21 g/cm^3^) was purchased from BASF Polyurethane Specialties (China) Co., Ltd. and was dried at 60 °C for 5 h before use. *N*,*N*-dimethyl formamide (DMF, 99.5%), tetrahydrofuran (THF, 99.0%), silicone oil, liquid paraffin, and ethylene glycol were purchased from Sinopharm Chemical Reagent Co. Ltd. Diiodomethane was purchased from Aladdin Industrial Corporation Co. Ltd. DuPont Krytox® GPL K103 lubricant oil was purchased from Miller-Stephenson (USA, LOT-K3268). RB aqueous solution was prepared by dissolving RB powders in DI water at a final concentration of 0.1 mg/mL. All chemicals were chemically pure-grade and were used as received. Air was used as the gas. Milli-Q DI water with a resistivity of 18.2 MΩ cm was used in all experiments.

### Fabrication and preparation of the GO/TPU composites and liquid-infused GO/TPU membrane

A schematic representation of the fabrication process of the GO/TPU composite membrane is shown in Fig. S1. First, graphite oxide was dispersed in DMF solvent (5 mg/mL) and exfoliated via ultrasonication for 2 h by using an intelligent ultrasonic processor (Ningbo Licheng Instrument Co. Ltd., China) with a power of 1000 W, thereby forming a uniform suspension of graphene oxide. Then, GO/TPU composites were prepared by a solution blending method followed by a heat curing process. Ten grams of TPU was dissolved in a mixture consisting of 15 mL of THF and 25 mL of DNF and then stirred in a magnetic stirrer at 60 °C until a homogeneous and transparent dispersion was formed. Subsequently, a well-dispersed suspension of GO/DMF was mixed with the solution of TPU under stirring for 2 h at 60 °C to obtain a uniform GO/TPU suspension with a ratio of GO to TPU ranging from 0.0 to 10.0 wt.%. The coagulated mixture was cured for 4 h at 40 °C and then for 8 h at 60 °C to completely remove the solution from the polymer matrix and immobilize the GO into the TPU matrix. Finally, GO/TPU samples were prepared by compression molding with a vacuum hot press (Vigor Machinery Co. Ltd., China). All samples were hot-pressed at 5 MPa at 150 °C for 10 min. TPU composites with 0 to 10 wt.% GO were prepared by the same procedure, and the resultant samples were designated TPU, GO/TPU-1, GO/TPU-2, GO/TPU-5, GO/TPU-8, and GO/TPU-10 according to the filler concentration. Photographs of the different TPU composites are shown in Fig. S2 in the Supporting Information. Liquid-infused membranes were generated by impregnating silicone oil, liquid paraffin, or Krytox 103 into porous membranes. They were prepared by dropping ~15 μL/cm^2^ gating liquid on the surfaces of porous membranes, and uniform coverage was achieved after a while.

### Material characterization

The structure of the TPU composites was determined by X-ray powder diffraction (XRD) (Rigaku Ultima IV, Japan) at a scanning rate of 4°/min and with a scanning angle ranging from 5 to 60°. The FTIR spectra of TPU and its composites were obtained using an FTIR spectrometer (Thermo Fisher FTIR) in attenuated total reflectance (ATR) mode. In all cases, the spectra were obtained within the wavenumber range of 4000-400 cm^−1^ at a scan rate of 4 cm^−1^.

### Surface characterization

A Hitachi S-4800 scanning electron microscope, operated at an acceleration voltage of 15 kV, was used to observe the morphology of the GO and GO/TPU composites. An atomic force microscope (Asylum Research, USA), operated in contact mode, was used to determine the thickness of GO. Fluorescent and bright-field optical images were obtained by an Olympus TH4-200 microscope. A digital camera (Nikon D750) was used to take photos and record videos.

### Stress-strain test and deformation test

Tensile tests were performed using an MTS Exceed 44.104 tensile testing machine under a constant displacement rate of 50 mm/min^−1^ at room temperature. The rectangular area of the dumbbell-shaped sample was 27.3 mm × 5.1 mm.

The Young’s moduli of the pure TPU and GO/TPU elastomeric membranes were determined by calculating the slopes of the stress-strain curves within 0–10% strain.

For the static deformation test, the porous membrane was prestretched and then sealed in two polymethyl methacrylate sheets to measure the critical pressures. For the dynamic deformation test, the porous membrane was assembled and sealed with several layers of 3M VHB tape and then fixed on the electric tensile rig fabricated by Shanghai Shigan Co. Ltd. to perform the stretching and releasing experiments. The adhesive agent Kejia KJ-770-90EP1 (purchased from Shenzhen Kejia Adhesive Material Co., Ltd) was applied to the membrane to enhance the adhesion between the membrane and 3M VHB tape.

### Wetting characterization

The contact angle, surface tension, and interfacial tension between different liquids were determined by a contact angle measurement system (OCA100, Data Physics). The CA measurements were performed through the sessile drop method by placing a drop of 5 μL on multiple areas of the surface of the sample. Surface tension and interfacial tension were determined by the pendant drop method. All mentioned values were an average of at least three measurements.

### Antifouling property measurements

The fouling resistance of the GO/TPU EPM and GO/TPU LGEPM membranes was tested by the passage of the RB solution through the membrane for 1 min for a total volume of 0.5 mL with a stationary interval of 30 s between every fouling cycle followed by a rinse with 5 mL of DI water. The concentration of the RB solution was 0.1 mg/ml.

### Transmembrane pressure measurements

The transmembrane properties of the GO/TPU EPM with and without gating liquids were determined by measuring the transmembrane pressure (Δ*P*) during the flow of gases and liquids. The measurement of Δ*P* was performed with wet/wet current output differential pressure transmitters (PX154-025DI) from OMEGA Engineering Inc. (Stamford). A multiporous membrane with 9 × 9 pores in a square shape (the average length is 50 mm, as shown in Fig. S1) was sealed in 3 M VHB tape. A Harvard Apparatus PHD ULTRA syringe pump was used in all experiments. A flow rate of 500 μL/min was used in the experiments shown in Figs. 3b, c, 4a–d, and 5c. For the gas–liquid separation experiments, air and DI water were pumped together to form a gas–liquid mixture at a flow rate of 200 μL/min. The separation efficiency was determined by comparing the volume of liquid obtained in the outlet with the volume of input liquid during a period of transporting gas/liquid mixture through the GO/TPU LGEPM.

## Supplementary information

Supporting information-clean version

Graphical abstract

Editorial summary
