# Supplementary material for: Highly stretchable and reliable graphene oxide-reinforced liquid gating membranes for tunable gas/liquid transport
Source: Microsyst Nanoeng. 2020 Jul 13;6:43. doi: 10.1038/s41378-020-0159-x (PMC8433400; doi:10.1038/s41378-020-0159-x)
Supplement: Supplementary file 1 — Supporting information-clean version [file 41378_2020_159_MOESM1_ESM.docx]

***Supplementary Information of***

**Highly Stretchable and Reliable Graphene Oxide-Reinforced Liquid Gating Membranes for Tunable Gas/Liquid Transport**

Wei Lv^1,#^, Zhizhi Sheng^2,3,#^, Yinglin Zhu^2^, Jing Liu^2^, Yi Lei^1^, Rongrong Zhang^2^, Xinyu Chen^2^, Xu Hou^1,2,3*^

^1^Department of Physics, Research Institute for Biomimetics and Soft Matter, Fujian Provincial Key Laboratory for Soft Materials Research, Jiujiang Research Institute, College of Physical Science and Technology, Xiamen University, Xiamen 361005, China

^2^State Key Laboratory of Physical Chemistry of Solid Surfaces, College of Chemistry and Chemical Engineering, Xiamen University, Xiamen 361005, China

^3^Collaborative Innovation Center of Chemistry for Energy Materials, Xiamen University, Xiamen 361005, China

# These authors contributed equally to this work.

* Correspondence: Xu Hou ([houx@xmu.edu.cn](mailto:houx@xmu.edu.cn))

1. **The fabrication process of graphene oxide-reinforced thermoplastic polyurethanes (TPU) liquid gating elastomeric porous membranes.**


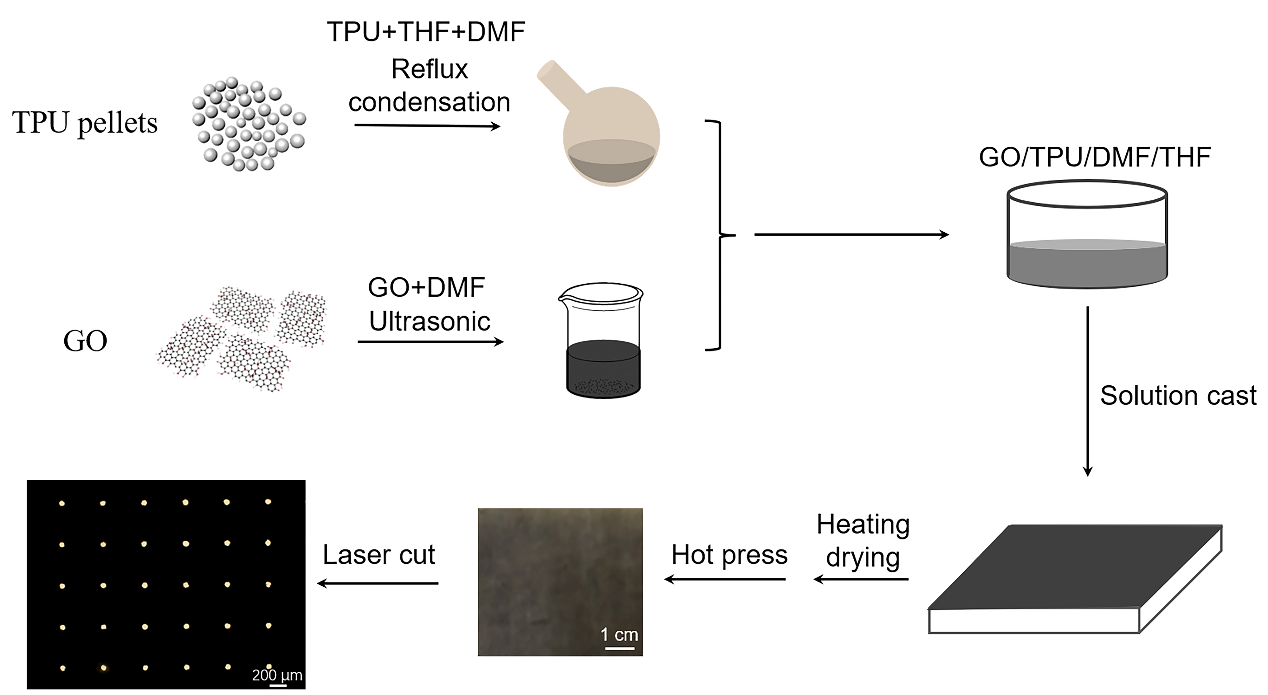


**Figure S1.** Schematic preparation process of graphene oxide-reinforced TPU liquid gating elastomeric porous membranes.

1. **The morphology and thickness characterization of graphene oxide (GO).**


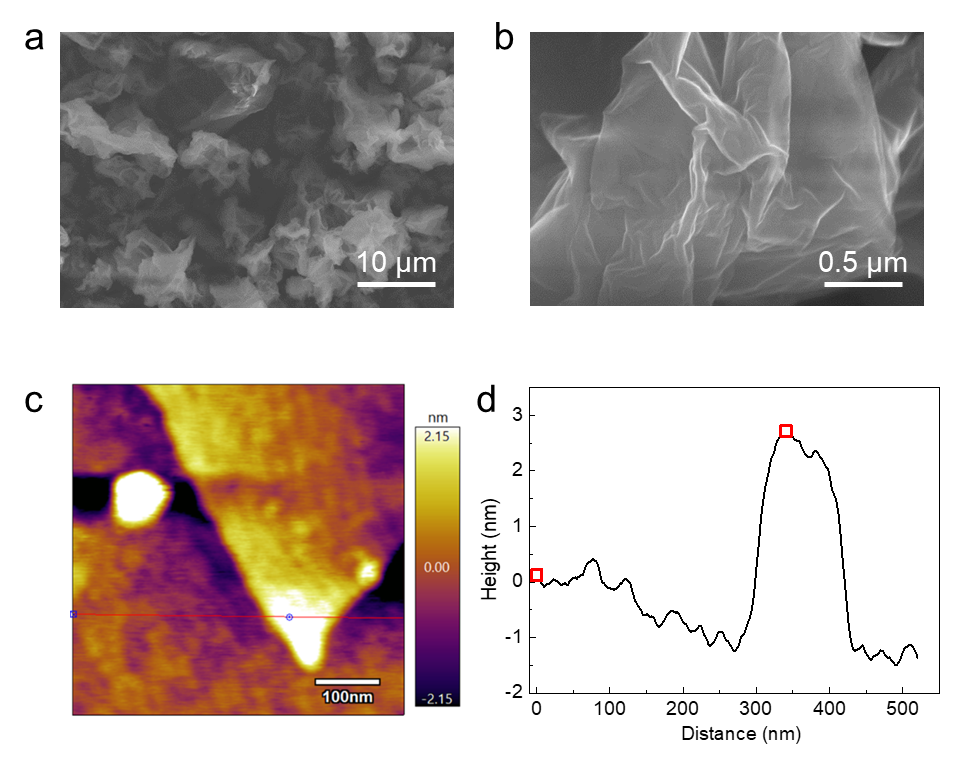


**Figure S**2. **a** SEM image of GO at lower magnification. **b** SEM image of GO at higher magnification. **c** The AFM image of GO (position of cross section in the image indicated by red, straight line). **d** The cross section (right) of GO. Typical thickness is 2.5 nm.

1. **The optical images of GO/TPU composites.**


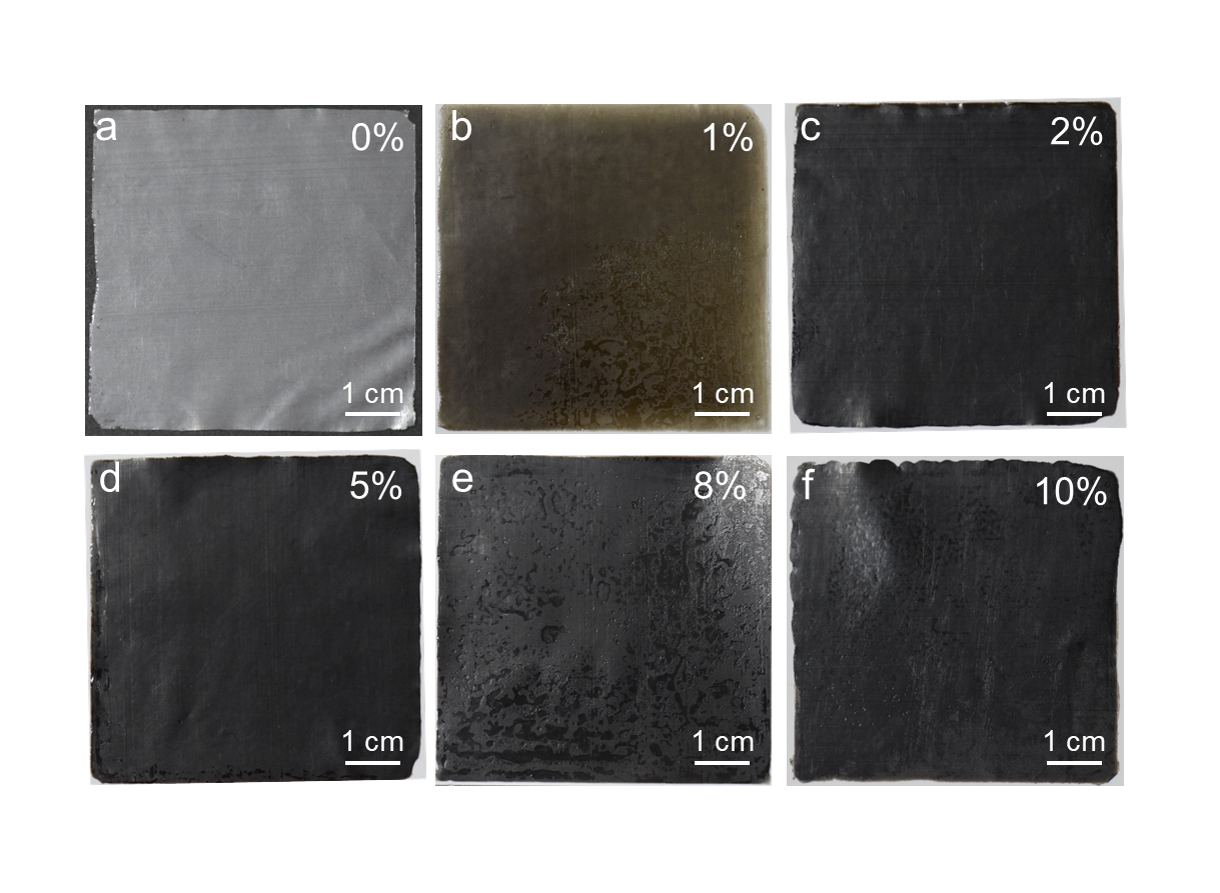


**Figure S3.** The optical images of GO/TPU composites with 0% (a), 1% (b), 2% (c), 5% (d), 8% (e), 10% (f) GO contents (weight percentage). As the filling content increases, the color of the composites gradually deepens.

1. **The Fourier Transform Infrared (FTIR) spectrum of GO.**


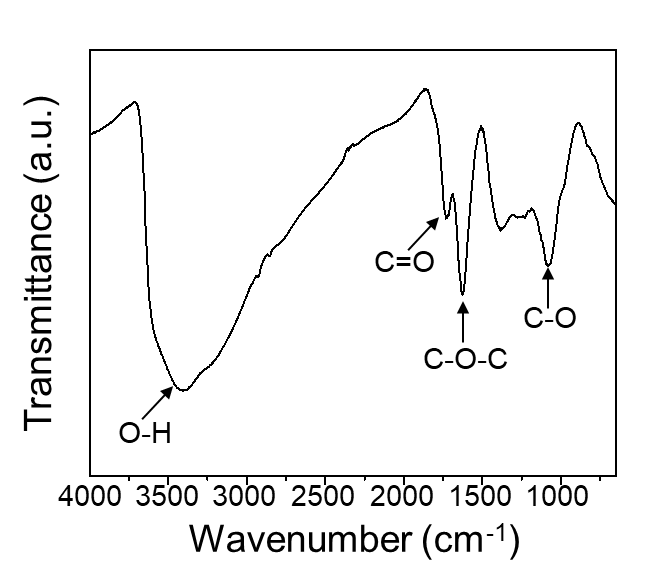


**Figure S4.** FTIR spectrum of GO. The peaks at O–H (3410 cm^-1^), C=O (1735 cm^-1^), C-O-C (1627 cm^-1^), and C–O (1079 cm^-1^) can be observed, which represent the oxygen-containing groups in GO^1^.

1. **The summary of d spacing and crystal size of GO/TPU composites.**

**Table S1.** The summary of d spacing and crystal size of the diffraction peaks in GO/TPU composites.

| Sample name | Peak 1 | | | | Peak 2 | | | |
| --- | --- | --- | --- | --- | --- | --- | --- | --- |
|  | Peak position (°) | d spacing (nm) | Plane index | Crystal size (nm) | Peak position (°) | d spacing (nm) | Plane index | Crystal size (nm) |
| TPU (0%) | NA | NA | NA | NA | 20.315 | 0.43678 | (110) | 10.1 |
| GO/TPU (1%) | NA | NA | NA | NA | 20.315 | 0.43678 | (110) | 11.1 |
| GO/TPU (2%) | 8.923 | 0.99026 | (002) | 12.6 | 20. 315 | 0.43678 | (110) | 9.4 |
| GO/TPU (5%) | 8.923 | 0.99026 | (002) | 8.6 | 20. 315 | 0.43678 | (110) | 7.2 |
| GO/TPU (8%) | 8.923 | 0.99026 | (002) | 10.2 | 20. 315 | 0.43678 | (110) | 9.4 |
| GO/TPU (10%) | 8.923 | 0.99026 | (002) | 10.4 | 20.315 | 0.43678 | (110) | 8.8 |

1. **The critical pressures for transporting gases and liquids at different flow rates.**


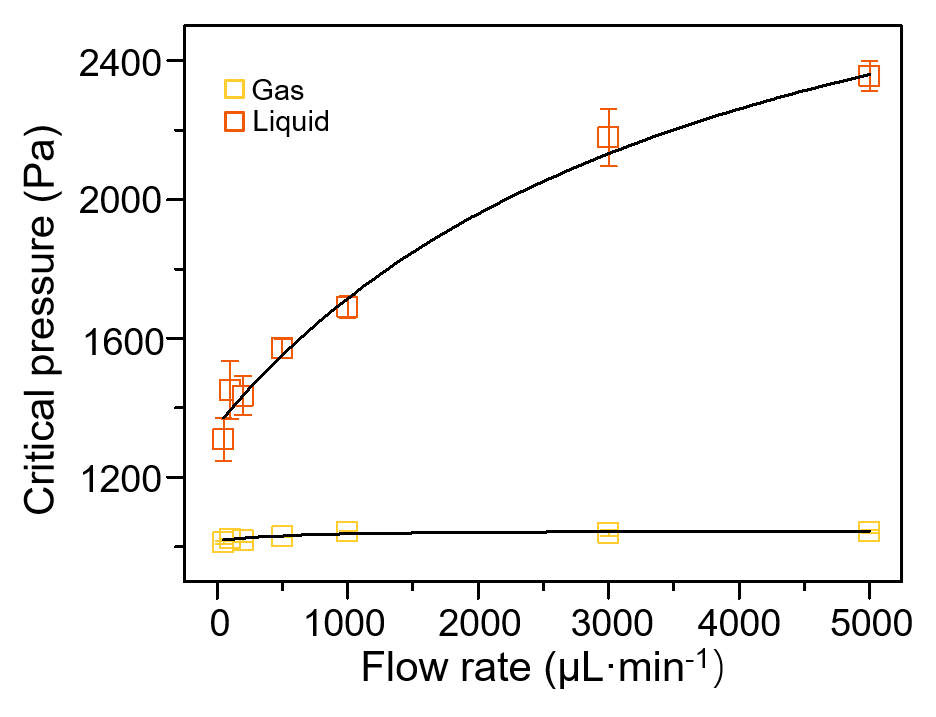


**Figure S5.** The critical pressures of gases and liquids flowing through the membrane at different flow rates. The increase in flow rate leads to an increase in the critical pressure.

1. **Equivalent stress distribution of 4 × 4 array micropore GO/TPU elastomeric porous membrane under uniaxial stretching**


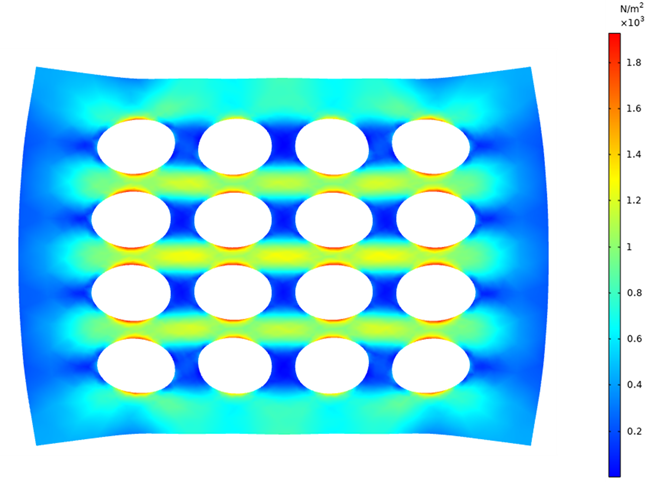


**Figure S6.** Equivalent stress distribution of 4 × 4 array micropore GO/TPU elastomeric porous membrane under uniaxial stretching. The force applied during the simulation was 500 Pa and the pore size was 64 μm.

1. **The sketch for tunable gas/liquid transport**


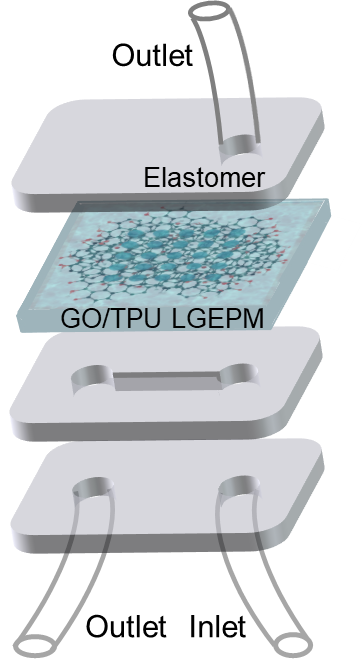


**Figure S7.** The sketch of the device for tunable gas/liquid transport. Elastomer is the 3M VHB tape. An adhesive agent (KeJia KJ-770-90EP1) was applied on both sides of the membrane to increase the adhesion between the membrane and 3M VHB tape.

**Reference**

1. Titelman, G.I. et al. Characteristics and microstructure of aqueous colloidal dispersions of graphite oxide. *Carbon* **43**, 641-649 (2005).
