# Supplementary figures and images for: Highly stretchable and reliable graphene oxide-reinforced liquid gating membranes for tunable gas/liquid transport
Source: Microsyst Nanoeng. 2020 Jul 13;6:43. doi: 10.1038/s41378-020-0159-x (PMC8433400; doi:10.1038/s41378-020-0159-x)

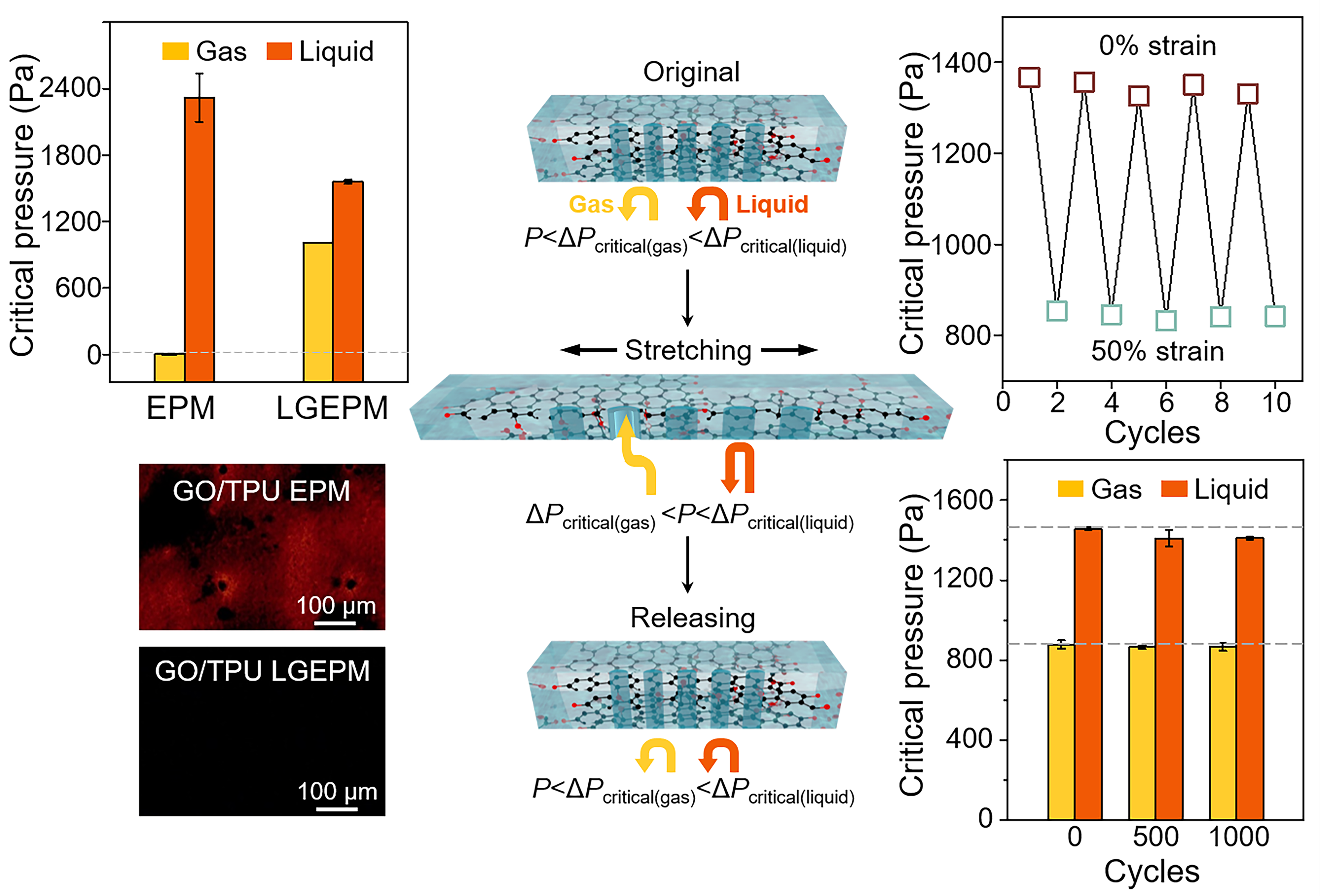

Supplement: Supplementary file 2 — Graphical abstract [file 41378_2020_159_MOESM2_ESM.tif]
