# Supplementary material for: Highly stretchable and reliable graphene oxide-reinforced liquid gating membranes for tunable gas/liquid transport
Source: Microsyst Nanoeng. 2020 Jul 13;6:43. doi: 10.1038/s41378-020-0159-x (PMC8433400; doi:10.1038/s41378-020-0159-x)
Supplement: Supplementary file 3 — Editorial summary [file 41378_2020_159_MOESM3_ESM.docx]

# *Microsystems & Nanoengineering*

Fluidics: Tuning into better performing membranes

Chinese scientists have developed a tunable membrane that is highly stretchable and could be used in a range of applications, including microfluidic devices, multiphase microreactors, and for particulate material synthesis. The ability to dynamically tune the transport behaviour of gases and liquids is critical for membrane applications. And although recent tunable elastomeric membrane designs offer flexibility and anti-fracture properties, they have low tensile strength, are unreliable, and suffer from fouling. Now, Xu Hou and colleagues from Xiamen University in China use a novel liquid gating technology to develop a membrane made from graphene oxide-reinforced thermoplastic polyurethane that can be dynamically tuned for a wide range of gases and liquids, while also exhibiting anti-fouling properties. Liquid gating technology which uses a capillary-stabilized functional liquid to form reversible gates inside the pores, shows prominent properties in controlling complex, selective, multiphase substance transport. The liquid gating membrane is adaptable to different length scales, pressures, and environments and could be used in multiphase separation, chemical reactions, and drug delivery.

Related article manuscript number: MICRONANO-01124R

Article title: Highly Stretchable and Reliable Graphene Oxide-Reinforced Liquid Gating Membranes for Tunable Gas/Liquid Transport

Corresponding author and affiliation/s: Xu Hou, Xiamen University, College of Chemistry and Chemical Engineering, Xiamen, Fujian, China; Xiamen University, College of Physical Science and Technology, Xiamen, Fujian, China.

**About your Editorial Summary — please read**

**Before approving this Editorial Summary, please carefully check that (1) the summary text lists the correct author(s) and (2) the spelling and order of all author names and affiliations are correct.**

This **Editorial Summary** is based on your manuscript that was recently accepted for publication in *Microsystems & Nanoengineering*. It provides a non-specialist audience with a synopsis of your key research outcomes and conclusions. This value-added service provided by Springer Nature is designed to raise interest in your research across the broader community.

Springer Nature will publish the summary on the journal’s website, and it will be freely available under a under the CC BY licence (Creative Commons Attribution v4.0 International Licence) (see the journal website for details). We encourage you to re-use the summary to bring attention to your research; for example, you can host it on your own website and share it via social-networking platforms. Please attribute the summary to *Microsystems & Nanoengineering* and your article (e.g. by providing a link to your article) and do not make derivatives.

Please note that to maximise the usefulness of these summaries they must follow several stringent guidelines:
-- Spelling, punctuation and style are set according to *Nature* editorial guidelines. As this summary is aimed at non-expert readers, some concepts and technical terms will be simplified.
-- Total length must be no more than 135 words. It is likely that not all points in the paper will be covered.
-- The first sentence must be no more than 280 characters, including spaces, to allow use on microblogging sites.
-- The headline must consist of a brief generic subject identifier followed by a short description. No more than 10 words in total.

Please contact the editorial office ([mems_nano@mail.ie.ac.cn](mailto:mems_nano@mail.ie.ac.cn)) immediately with corrections should you find any factual errors in this Editorial Summary.
